# The Effect of Ivabradine on the Heart Rate and Sympathovagal Balance in Postural Tachycardia Syndrome Patients

**DOI:** 10.5041/RMMJ.10213

**Published:** 2015-07-30

**Authors:** Merav Barzilai, Giris Jacob

**Affiliations:** 1 J. Recanati Autonomic Dysfunction Center, Tel Aviv “Sourasky” Medical Center, Tel Aviv, Israel; 2 Medicine F, Tel Aviv “Sourasky” Medical Center, Tel Aviv, Israel; 3 Faculty of Medicine, Tel Aviv University, Tel Aviv, Israel

**Keywords:** *I*_f_ channel blocker, postural tachycardia syndrome, orthostatic intolerance, sympathovagal balance

## Abstract

**Background:**

Postural tachycardia syndrome (POTS) is a common form of chronic orthostatic intolerance. The remarkable increase in heart rate (HR) upon standing is the hallmark of this syndrome. Treatment of POTS patients is challenging and includes drugs that slow the HR. Ivabradine is a selective I*_f_* channel blocker designed to slow the HR, as an anti-anginal agent. In view of its ability to slow the HR, we posited that ivabradine may be an ideal medication for treating POTS patients. This report provides the results of an investigation in which we studied ivabradine’s effect on the hemodynamics and sympathovagal balance in POTS patients.

**Methods:**

An open-label trial, without a placebo control, was performed in eight patients with POTS of two years’ standing. Characterization of symptoms, hemodynamics, autonomic function tests, and HR and blood pressure (BP) variability were determined while patients were in a supine position and during a 20-minute head-up tilt before and after a single oral dose of 7.5 mg ivabradine.

**Results:**

Ivabradine slowed the HR of POTS patients at rest by 4±1 bpm (*P*<0.05). During a 5-minute head-up tilt, the HR decreased from 118±4 bpm to 101±5 bpm (*P*<0.01). Ivabradine did not affect the BP when patients were at rest in a supine position or in head-up tilt position. Cardiovascular vagal and sympathetic tone, extrapolated from the time and frequency domains of the HR and BP variability, were also not affected by ivabradine.

**Conclusions:**

Ivabradine is an effective drug for slowing the HR of POTS patients at rest and during tilting, without producing significant adverse effects. Moreover, ivabradine exerts its effects without influencing the sympathovagal balance.

## INTRODUCTION

Postural tachycardia syndrome (POTS) is a common form of chronic orthostatic intolerance that occurs on standing and is eventually relieved by lying down or sitting. The syndrome is characterized by a constellation of orthostatic symptoms (which include lightheadedness, dimming of vision, confusion, fatigue, exercise intolerance, and anxiety) and clinical signs (which include a bluish-red skin and edema in dependent limbs). The main physical finding in POTS is the dramatic remarkable increase in heart rate (HR) upon standing (>30 beats per minute (bpm)) without an appreciable decrease in blood pressure (BP). Most POTS patients are women, and the age of presentation is usually between the ages of 15 and 50 years. The quality of life of POTS patients can be substantially reduced to a debilitating level.^1^–^4^

This syndrome is thought to be a primary adrenergic dysfunction. Most POTS patients have elevated plasma norepinephrine concentrations, hypovolemia, and excessive pooling of blood in the legs while standing.^5^,^6^ Some studies have shown that sympathetic denervation, especially of the veins in the legs, may be the underlying mechanism of the disease: such denervation results in blood pooling upon standing with reductions in the end-diastolic ventricular volume, and reflex tachycardia. Some POTS patients can present with idiopathic hypovolemia with or without a renin and aldosterone disorder. Other possible causes of POTS are a central hyperadrenergic state, which has a genetic predisposition, and indolent mastocytosis.^7^–^14^

Secondary POTS can occur in conditions that affect the peripheral autonomic nervous system such as diabetes mellitus, amyloidosis, sarcoidosis, and paraneoplastic syndromes, or following chemotherapy.^2^,^3^,^9^

Management of POTS is multifaceted and includes discontinuation of any medications that could contribute to orthostatic intolerance, such as α-adrenoceptor antagonists, diuretics, cGMP-specific phosphodiesterase type 5 inhibitors (sildenafil), and organic nitrates and nitrites, along with identifying those conditions that may cause secondary POTS.

Treating POTS patients comprises non-pharmacological and pharmacological interventions. Conservative measures for patients with mild POTS symptoms include plasma volume expansion and increasing dietary salt and fluid intake. Pharmacotherapy is needed for patients with moderate to severe POTS, and, currently, no drug has been officially approved by the US Food and Drug Administration (FDA) for treating POTS. In fact, most drugs that are given to POTS patients are usually prescribed “off label” and include fludrocortisone, desmopressin, and, sometimes, erythropoietin. Other drugs which can also be therapeutically beneficial in patients with POTS are (1) drugs that increase peripheral vascular resistance such as midodrine, a prodrug whose active metabolite is an α-adrenoceptor antagonist, and somatostatin analogs; (2) drugs that can modify the central and peripheral activity of the sympathetic nervous system such as β-adrenoceptor antagonists (mainly propranolol), serotonin and norepinephrine uptake inhibitors, and combined α- and β-adrenoceptor antagonists; and (3) drugs that reduce the sympathetic tone such as clonidine.^2^,^3^,^9^

Treatment of POTS is challenging due to the potential for adverse effects from the abovementioned drugs and the limited effectiveness of non-pharmacologic treatments. Many POTS patients have difficulty achieving adequate control of their symptoms. In addition, none of the abovementioned medications are tailored specifically to blunt the increase in HR that underlies the many symptoms of POTS.

There is some anecdotal evidence that some POTS patients can be successfully treated with ivabradine, an anti-anginal agent designed to slow the HR.^15^–^26^ Ivabradine is a selective antagonist of the I*_f_* channel, an ionic current that determines the slope of diastolic depolarization (phase IV action potential). Accordingly, ivabradine controls the time interval between successive action potentials and the HR. Ivabradine also reduces the firing rate of pacemaker cells in the sinoatrial (SA) node, where it mainly influences the intrinsic HR at concentrations that do not affect other cardiac currents, and has no negative inotropic or lusitropic effects.^17^,^27^–^32^ In view of its ability to slow the HR without affecting other cardiovascular functions, we posited that ivabradine may be an ideal medication for treating POTS patients. We report herein on the results of an investigation in which the effect of ivabradine on the hemodynamics and sympathovagal balance of POTS patients was studied.

## METHODS

The investigation was an open-label trial without placebo control. It was approved by the Tel Aviv Sourasky Medical Center Institutional Review Board and was performed according to the principles of the Declaration of Helsinki. The study was done in the J. Recanati Autonomic Dysfunction Center, Department of Internal Medicine F, Tel Aviv “Sourasky” Medical Center, Tel Aviv, Israel. After being provided with an explanation of the purpose, nature, and potential risks of the investigation, each recruited patient signed a consent form.

### Subjects

Inclusion criteria for the investigation were POTS patients with orthostatic intolerance for at least six months; increased HR of at least 30 bpm without a concomitant decrease in BP of more than 20/10 mmHg within 10 minutes of assuming a standing position or during a head-up tilt on at least three separate occasions; and the absence of any disease that could account for symptoms of orthostatic intolerance. Patients with orthostatic intolerance were excluded from the investigation if they were smokers; were pregnant; had an uncontrolled thyroid or adrenal disorder; had a history of a systemic illness that could influence autonomic function, such as diabetes mellitus or *s*ystemic lupus erythematosus, cardiovascular disease, or drug or alcohol abuse; or had taken any drug that was metabolized by cytochrome P450 (CYP3A4) during the last 72 hours.

Study participants were requested to stop taking their medications and not drink caffeine-containing beverages, such as coffee, tea, or cola drinks, and caffeine-containing foods, such as chocolate, for at least 24 hours prior to testing. The participants were also asked to complete an autonomic nervous system questionnaire in order to obtain information on the type and extent of their POTS symptoms before and after testing. Anamnesis, a physical examination, and electrocardiography (ECG) were performed on all participants prior to testing.

### Experimental Protocol

All measurements were made in each participant between 08.00 and 11.00 a.m. in a quiet, darkened, and air-conditioned room with an ambient temperature of ~24°C. The experimental protocol comprised six phases during which the BP and HR were continuously monitored. First, baseline measurements of the arterial BP using a finger plethysmograph (Nexfin, BMEYE, Amsterdam, Netherlands), HR using a three-lead ECG, and respiratory rate (RR) of each participant were made. Second, each participant rested in a supine position on a tilt table for 30 minutes. Third, the RR and BP were sampled for eight minutes by a 500 Hz A/D converter whose output was transferred to a standard personal computer using a specialized software package. The data on HR and BP variability (HRV and BPV, respectively) from each participant were analyzed off-line using a locally developed software package. Baroreflex sensitivity of each participant was calculated from the time-domain of the beat-to-beat HR and BP. Fourth, standard autonomic testing of the parasympathetic and sympathetic nervous systems were performed in each participant. Specifically, testing of the parasympathetic nervous system comprised the Valsalva maneuver and deep breathing, and testing of the sympathetic nervous system comprised a static hand grip for three minutes, hyperventilation for one minute, and immersion of the hand in ice water for one minute. Fifth, the patient was then head-up tilted at 70° for 20 minutes. Finally, the patient was instructed to rest in a supine position on a tilt table for 30 minutes. This protocol was repeated 60–80 minutes after administering a single oral dose of 7.5 mg ivabradine.

### Pharmacology of Ivabradine

Ivabradine was originally developed for the treatment of chronic stable angina pectoris. Ivabradine blocks the I*_f_* channel (funny channel, HCN2/4 channel), which is a mixed Na^+^–K^+^ inward current that causes hyperpolarization of the membrane and is highly expressed in the SA node and atrioventricular node. This channel is typically controlled by the sympathetic nervous system. The sympathetic and parasympathetic nervous systems cause an increase and a decrease, respectively, in the Na^+^ inward current and results in either tachycardia or bradycardia. Currently, ivabradine is approved for use in Europe only for anginal syndromes and inappropriate sinus tachycardia syndrome.

The pharmacokinetics and pharmacodynamics of ivabradine have been extensively studied in animals and humans. Its bioavailability is 40%, and its elimination half-time is about two hours. On first pass, approximately 50% of the drug is metabolized by CYP3A4. Its protein-binding capacity is approximately 70%, and it is eliminated by the kidneys with preserved partial activity of its metabolite. Dose adjustment is not needed for patients whose glomerular filtration rate is less than 15 mL/min. Its C_max_ is 8.8 ng/mL, and its t_max_ is 0.9 hours (45–90 minutes) for an oral dose of 5 mg. Maximal HR control is achieved by a 20 mg oral dose. In this study, we used a single oral dose of 7.5 mg for safety purposes. The main adverse effects of ivabradine are luminous phenomena (mainly a sensation of enhanced brightness with a fully maintained visual field), sinus bradycardia, first-degree atrioventricular blocks, ventricular extra systoles, dizziness, and/or blurred vision. Ivabradine is contraindicated in sick sinus syndrome and should not be taken concomitantly with CYP3A4 inhibitors.

### Statistical Analysis

All data were analyzed using Microsoft Office Excel and Prism version 5.5 (GraphPad Software Inc., La Jolla, CA, USA). Results are presented as mean ± standard error of the mean, and statistical significance was set at 5%. Parametric continuous data were analyzed using a paired *t* test. Non-parametric continuous data were analyzed using the Mann–Whitney *U* test. The HRV and BPV were extracted from the time and frequency domains of the beat-to-beat systolic BP and RR intervals using a locally developed software package.

## RESULTS

In one year, we were able to recruit eight patients who met our inclusion criteria. Their general characteristics are depicted in Table 1. Three subjects were taking fludrocortisone, and six subjects were taking propranolol, the β-adrenoceptor antagonist (mean dose 17±2 mg/day) at the time of recruitment. Propranolol administration was gradually discontinued before the day of testing.

**Table 1:** General Characteristics of the Eight Study Participants.

Characteristic	Detail
Age	31±3 years
Female/Male	6/2
Body Mass Index	27±1.5 kg/cm^2^
POTS Duration	2.6±1 years (median: 2 years)

POTS, postural tachycardia syndrome.

Patients reported that a single oral dose of 7.5 mg ivabradine attenuated the main orthostatic symptoms, such as dizziness, blurred vision, and palpitations. None of the participants reported adverse effects that were related to the test drug.

Ivabradine decreased the resting HR by 4±1 bpm. After tilting for five minutes, the HR significantly decreased (*P*<0.01) from 118±4 bpm to 101±5 bpm. Interestingly, ivabradine did not change the BP when the subject was resting in a supine position and during the head-up tilt (Table 2).

**Table 2:** Hemodynamic and Indices of Autonomic Function Before and After Ivabradine.

Parameter	Baseline		Ivabradine	
	Rest	Tilt	Rest	Tilt
HR (bpm)	70±3	118±4	66±2*	101±5*
Systolic BP (mmHg)	113±5	114±4	114±3	115±4
Diastolic BP (mmHg)	68±3	75±3	67±3	74±4
Valsalva ratio	1.43±0.1	-	1.52±0.2	-
RMSSD	35±4	16±2	28±6	18±2
L*f*_rri_	715±160	470±180	790±150	390±120
H*f*_rri_	790±120	180±80	840±140	210±90
L*f*_rri_/H*f*_rri_ ratio	1.15±0.2	3.9±1.0	0.95±0.1	4.1±1.2
L*f*_BP_	1.5±0.6	5.1±1.2	1.6±0.5	4.7±1.0

* Significant differences between the baseline and the tilt.

BP, blood pressure; bpm, beats per minute; H*f*_rri_, RR intervals high-frequency domain; HR, heart rate; L*f*_BP_, BP low-frequency domain; L*f*_rri_, RR intervals low-frequency domain; RMSSD, root mean square of the successive differences.

Ivabradine did not affect the cardiovascular vagal tone indices: Valsalva’s ratio (maximal increase (phase II)/maximal decrease (phase IV) in HR along the maneuver), time-domain analysis of the HR variability root mean square of the successive differences (RMSSD), and the high-frequency domain of RR intervals (H*f*_rri_). The funny channel blocker did not influence cardiovascular sympathetic tone, as measured by the indices of the cardiovascular control of HR and systolic BP, namely the low-frequency domain of RR interval and systolic BP changes (L*f*_rri_ and L*f*_BP_, respectively) (Table 2). Ivabradine also did not change the L*f*_rri_/H*f*_rri_ ratio during rest and tilt, a measure of the sympathovagal balance of HR (Table 2).

## DISCUSSION

The results of this investigation showed that a single oral dose of 7.5 mg ivabradine significantly slowed the HR of POTS patients at rest and during tilting without any relevant adverse effects. Moreover, this dose of ivabradine had no effect on the indices of cardiovascular autonomic nervous system activity, especially the HRV.

Ivabradine is a pure HR-slowing agent, whose pharmacological target is the SA node. Hence, it is not altogether surprising that ivabradine slows the HR in POTS patients. This slowing of the HR can also be achieved following administration of β-adrenoceptor antagonists.^9^ Since this slowing of the HR is associated with adverse effects such as fatigue, sleep disorders, and impotence, many POTS patients frequently discontinue treatment with β-adrenoceptor antagonists.

There are anecdotal reports that describe a therapeutically beneficial effect of ivabradine on POTS symptoms and HR reduction in POTS patients. Sutton et al. recently reported that ivabradine prevented syncope in patients with vasovagal syncope that is preceded by tachycardia.^33^,^34^ McDonald et al. reported their clinical observations on the efficacy, drug tolerance, and adverse effects of ivabradine in 22 POTS patients.^35^ They found that ivabradine was well tolerated in most patients, and only a few adverse effects were reported. Six of these 22 patients discontinued the medication after 15 weeks due to the lack of a therapeutic benefit.

Our knowledge of the I*_f_* channel, located in the SA node, is primarily based on animal studies. The activity of this channel is regulated by the autonomic nervous system (ANS).^17^ The sympathetic arm of the ANS activates the β_1_-adrenoreceptor; its second messenger, cyclic AMP, increases I*_f_* channel conductance. This increased conductance permits more Na^+^ ions to move into the cells of the SA node. The HR increases due to the reduced polarity (phase IV of the action potential) of these cells. Parasympathetic or vagal tone of the heart is facilitated by M2 muscarinic receptors in the SA node. Stimulation of these receptors results in bradycardia by increasing the polarity of these cells in the SA node. Therefore, the sympathovagal balance of the SA node should affect I*_f_* channel activity. We found that ivabradine did not change the sympathovagal balance, despite significant slowing of the HR. We also expected the indices of the vagal tone, namely the RMSSD and the H*f*_rri_, to increase with the simultaneous decrease in sympathetic tone. These results are therefore unexpected and we have no precise explanation. However, possible explanations include (1) the low dose of ivabradine that was given to our study patients, (2) that ivabradine was given as an acute single oral dose before testing, and testing was not done in POTS patients who had been using ivabradine for a prolonged period, (3) the early sampling of the ECG and BP (time-dependent effect), or (4) that the pharmacological action of ivabradine exerted on the funny channel may have spared the “outcome” of sympathovagal tone.

Joannides et al. recently reported that acute administration of ivabradine has no effect on ANS activity and function in healthy subjects, as measured by HRV analysis, despite slowing of the HR during rest and exercise.^36^ They also reported no evidence of a depressant effect on cardiac function. Nerla et al. also reported that the ivabradine-induced increase in vagal cardiovascular tone, in syncope patients, was very modest and less than that observed in patients who were given a β-adrenoceptor antagonist.^37^

## CONCLUSIONS

Ivabradine is a selective blocker of the I*_f_* channel in cells of the SA node and is effective in slowing the HR of POTS patients at rest and during tilting, without producing significant adverse effects. Moreover, ivabradine exerts its effects without influencing the sympathovagal balance. In view of the limited information on the therapeutic benefits of ivabradine in POTS patients, there is a need to carry out additional investigations on its clinical effectiveness in a large group of POTS patients in a randomized double-blind placebo-controlled cross-over trial.

## Abbreviations

ANS: autonomic nervous system
BP: blood pressure
BPV: blood pressure variability
ECG: electrocardiography
HR: heart rate
HRV: heart rate variability
POTS: postural tachycardia syndrome
RMSSD: root mean square of the successive differences
SA: sinoatrial.
